# Enhancing plant resilience: arbuscular mycorrhizal fungi’s role in alleviating drought stress in vegetation concrete

**DOI:** 10.3389/fpls.2024.1401050

**Published:** 2024-06-21

**Authors:** Shiwei Guo, Lu Xia, Dong Xia, Mingyi Li, Wennian Xu, Liming Liu

**Affiliations:** ^1^ Key Laboratory of Geological Hazards on Three Gorges Reservoir Area (China Three Gorges University), Ministry of Education, Yichang, Hubei, China; ^2^ College of Civil Engineering & Architecture, China Three Gorges University, Yichang, Hubei, China; ^3^ Hubei Provincial Engineering Research Center of Cement-based Ecological Restoration Technology (China Three Gorges University), Yichang, Hubei, China; ^4^ College of Hydraulic & Environmental Engineering, China Three Gorges University, Yichang, China

**Keywords:** vegetation concrete, drought stress, arbuscular mycorrhizal fungi, photosynthetic physiology, antioxidant enzyme activity, osmotic adjustment substances, drought resistance

## Abstract

**Introduction:**

Drought stress usually inhibits plant growth, which may increase the difficulty of greening slopes.

**Methods:**

In this study, we systematically investigated the effects of arbuscular mycorrhizal (AM) fungi on the growth and drought tolerance of two plant species, *Festuca elata* and *Cassia glauca*, in a vegetation concrete environment by exogenously inoculating AM fungi and setting three drought levels: well water, moderate drought and severe drought. The results showed that plant growth was significantly inhibited under drought stress; however, AM fungi inoculation significantly promoted plant height, root length, and above- and belowground biomass in these two plant species.

**Results:**

Compared with, those in the CK treatment, the greatest increases in the net photosynthesis rate, stomatal conductance and transpiration rate in the AM treatment group were 36.72%, 210.08%, and 66.41%, respectively. Moreover, inoculation with AM fungi increased plant superoxide dismutase and catalase activities by 4.70–150.73% and 9.10–95.70%, respectively, and reduced leaf malondialdehyde content by 2.79–55.01%, which alleviated the damage caused by oxidative stress. These effects alleviated the damage caused by oxidative stress and increased the content of soluble sugars and soluble proteins in plant leaves by 1.52–65.44% and 4.67–97.54%, respectively, which further increased the drought adaptability of plants. However, inoculation with AM fungi had different effects on different plants.

**Conclusion:**

In summary, this study demonstrated that the inoculation of AM fungi in vegetation concrete environments can significantly increase plant growth and drought tolerance. The plants that formed a symbiotic structure with AM fungi had a larger root uptake area, greater water uptake capacity, and greater photosynthesis and gas exchange efficiency. In addition, AM fungi inoculation further increased the drought adaptability of the plants by increasing their antioxidant enzyme activity and regulating their metabolite content. These findings are highly important for promoting plant growth and increasing drought tolerance under drought conditions, especially for potential practical applications in areas such as slope protection, and provide useful references for future ecological engineering and sustainable development.

## 1 Introduction

Human-induced climate change, as outlined in the IPCC’s 2023 Synthesis Report (IPCC, 2023), has begun altering weather patterns and exacerbating climate extremes worldwide. Failure to adapt to these changes poses significant risks to both human societies and ecological systems (Dodd et al., 2023). Notably, terrestrial carbon sinks play a crucial role in mitigating anthropogenic emissions, but the degradation of vegetation has increased the frequency of extreme weather events (Friedlingstein et al., 2022; Li et al., 2023). With terrestrial vegetation absorbing a substantial amount of carbon annually through photosynthesis, enhancing vegetation quality and expanding vegetated areas offer a viable means of offsetting carbon emissions (Sha et al., 2022). However, the creation of slopes through engineering excavation, particularly for large-scale hydraulic projects and road construction, has led to adverse impacts on surrounding ecosystems, including soil erosion and vegetation loss (Chen et al., 2023). While these slopes offer potential for increasing global vegetation coverage and mitigating carbon emissions, many of these slopes are characterised by rocky surfaces that are unsuitable for plant growth. Moreover, rainfall exacerbates soil erosion and nutrient loss, further hindering vegetation establishment (Xerdiman et al., 2022). To address these challenges, it is crucial to enhance the drought resistance of slope plants and mitigate soil erosion caused by rainfall. Traditional slope protection techniques usually control erosion, but they often do little to help plant growth (Yang et al., 2019). In contrast, vegetation concrete technology offers a promising solution by providing a stable substrate for vegetation growth on rocky slopes. By laying metal wire mesh and anchors to secure the substrate and incorporating cement to enhance resistance to scouring, vegetation concrete creates favour conditions for slope vegetation (Shu et al., 2022). This technology is increasingly utilised in various infrastructure projects, including water conservation initiatives, highways, and railways (Faiz et al., 2022). In conclusion, while traditional slope protection methods have limitations in facilitating vegetation growth, vegetation concrete technology represents an innovative approach for ecological restoration and vegetation improvement on slopes. By addressing the shortcomings of conventional techniques and providing conducive conditions for plant establishment, vegetation concrete offers promising prospects for enhancing slope resilience and mitigating the impacts of climate change. However, in recent years, most of the studies on vegetation concrete have focused on heavy rainfall, seismic zones and alpine areas, and relevant studies have been carried out on environmental factors such as resistance to scouring, earthquakes, and freezing and thawing, but studies on arid zones are not yet available (Qu et al., 2020; Xia et al., 2023; Yang Y S et al., 2023).

In recent years, the increasing impacts of climate change have triggered significant shifts in precipitation and temperature patterns across various regions worldwide (Cui et al., 2022; Liu K et al., 2023; Jang et al., 2022; Tang et al., 2023). The exacerbation of temperature extremes and drought occurrences contributes to heightened mortality rates in numerous plant communities globally (Hammond et al., 2022; Sadhukhan et al., 2022; Sanchez-Martinez et al., 2023). In the ecological restoration of slopes, the steep slopes lead to the formation of surface runoff for the sufficient infiltration of water on the slopes, as well as a strong evapotranspiration effect, resulting in slope plants being susceptible to drought stress. Drought represents a significant stressor to plant physiology, growth, and reproductive functions, leading to compromised photosynthetic efficiency, slowed growth rates, and observable manifestations such as decreased leaf density, shortened stems, and reduced biomass (Sanchez-Martinez et al., 2023). Moreover, drought conditions trigger an overproduction of reactive oxygen species within plants, resulting in cellular-level structural impairments. Prolonged drought periods can even culminate in cellular death and plant wilting (Sheteiwy et al., 2021a). These challenges are particularly pronounced in slope ecological restoration engineering, where vegetation faces heightened vulnerability to water stress. Despite these challenges, recent research efforts have focused on mitigating the effects of drought on ecological restoration on slopes through the development of new water-saving irrigation techniques to rationalise irrigation, the addition of water-holding agents and other means (Zhang et al., 2019; Xerdiman et al., 2022). However, these studies tend to ignore their inherent limitations. Notably the adoption of water-saving techniques and water retention agents usually requires high maintenance costs or significant resource inputs. Although these methods may reduce water use to some extent, they have yet to significantly improve the sustainability and resilience of slope ecological restoration projects. Therefore, the above methods are currently not effective in addressing the long-term viability and drought tolerance of slope ecosystems. Moving forwards, there is a critical need to reassess the predominant reliance on water-saving irrigation technologies and explore alternative strategies to bolster drought resilience in slope ecological restoration engineering. Integrating ecological principles, such as leveraging plant microbe interactions and ecosystem-based approaches, could offer promising avenues for enhancing the sustainability and resilience of slope ecosystems in the face of escalating climate challenges. By embracing a holistic and ecologically driven approach, future research endeavours can strive towards more effective and enduring solutions for mitigating the impacts of drought on slope ecological restoration engineering projects.

Arbuscular mycorrhizal (AM) fungi in the soil play a key role in addressing the challenges of drought (Huang et al., 2021). AM fungi form symbiotic relationships with approximately 80% of terrestrial plants and can significantly increase photosynthetic efficiency, biomass, and enzyme activity, among other factors (Cheng et al., 2021; Yang Y M et al., 2023). In addition, AM fungi can also enhance mineral nutrient uptake to enhance root branching and root length, promote the synthesis of plant hormones, influence gene expression, and alter secondary metabolism (antioxidants and phenolic and polyamine metabolism); at the same time, AM fungi secrete globulins that sequester heavy metals, increasing plant resistance to abiotic stressors (Sheteiwy et al., 2021b; Ahmed et al., 2023; In ‘t Zandt et al., 2023; Sheteiwy et al., 2023). Through their extraroot mycelia, AM fungi can enter soil pores and increase plants’ ability to absorb water and nutrients, thereby fostering improved plant growth (Cheng et al., 2021; Huang et al., 2021). In addition, AM fungi facilitate the effective control of the internal water balance, regulate plant stomatal behaviour, improve water utilisation efficiency, and mitigate the adverse effects of water deficit on plants (Duan et al., 2021).

AM fungi help promote plant growth to improve drought resistance, but they have been used less often in slope protection and for the first time in vegetated concrete technology. Additionally, considering the slightly alkaline nature of the specific environment of vegetation concrete and its differences from natural soils, there is a need to study in-depth the mechanisms involved in the use of AM fungi to improve the drought tolerance of plants. The aim of this study was to investigate the effect of AM fungi on plant growth physiology and drought resistance under drought stress conditions by the exogenous inoculation of AM fungi using vegetated concrete as the test soil and to investigate the effectiveness of AM fungi in improving the drought resistance of slope plants. This study aimed to provide a more scientific foundation for addressing challenges related to drought in slope greening processes.

## 2 Materials and methods

### 2.1 Experimental design

The test strains used in this study were *Funneliformis mosseae* (FM) and *Rhizophagus intraradices* (RI), both of which were supplied by the Institute of Mycorrhizal Biotechnology, Qingdao Agricultural University. The mycorrhizal inoculum contained spores (20–30 spores/g), root segments, mycelia and amplification substrates (Sand and gravel mixtures used as plant soil in the expansion of AM fungi, which will contain the presence of AM fungal spores). A total of 24 treatments were used in the experiment, including four inoculation methods, three drought levels, and two plant species. There were 12 replicates for each treatment, 6 for 30-day sampling, and 6 for 60-day sampling. Drought stress was initiated four weeks after sowing, and for pot cultivation, a weight-based approach was used to maintain the water gradient. Weighing was conducted every day at 6 PM, and the soil moisture content was regulated through manual irrigation. Before soil drought began, the potted soil water maintained 75% of the maximum field water-holding capacity (corresponding to WW). Drought treatments began in the fifth week, and the drought levels included well-watered (WW, 65–75% field capacity), moderate drought (MD, 45–55% field capacity) treatments, and severe drought (SD, 30–40% field capacity). The inoculation methods included a blank control (CK), *Rhizophagus intraradices* alone (RI), *Funneliformis mosseae* alone (FM), and mixed inoculation with FM+RI (FR). CK, FM and RI also needed to be inoculated with sterilising agents from uninoculated strains. The pot calibre was 16 cm, the base diameter was 12 cm, the height was 12cm, and each pot was filled with 2000 g of soil and 50 seeds. First, the configured vegetation concrete substrate was packed into the pots for approximately 6cm as the bottom layer, 400 g of vegetation concrete substrate mixed with mycorrhizal agent was added to the pots as the inoculation layer; the seeds were evenly sown on the surface of the inoculation layer, and, finally the substrate was covered with 2 cm as the surface layer (the total weight of the soil was kept at 2000 g throughout the process). The specific inoculation dose is shown in **Table 1**. In addition, sawdust (organic matter) and habitat substrate amendments were added. The specific configuration method used was based on the national industry standard “Technical Code for Eco-restoration of Vegetation Concrete on Steep Slope of Hydropower Projects” (National Energy Administration of the People’s Republic of China, 2019). The inoculum quantity was determined according to the Chinese gram-negative Bacterial Corpus (BGC) (Iffis et al., 2017). The experiments were carried out in a greenhouse in front of the Geology Building of Three Gorges University in Yichang, Hubei Province, China. (111°18′17″E, 30°43′46″N).

**Table 1:** AM fungi agent inoculation method.

Host plants	Exogenous inoculation treatments of AM fungi(g)			
	CK	RI	FM	FR
*Festuca elata*	inactivated RI:25 inactivated FM:25	RI:25 inactivated FM:25	inactivated RI:25 FM:25	RI:25 FM:25
*Cassia glauca*				

### 2.2 Data analysis

Plant height was determined via direct measurement with a ruler at 30 and 60 days after the onset of drought stress. Three representative and equally vigorous plants were selected from the designated pots for each time point, resulting in a total of 9 replicates. Biomass was determined by separating the aboveground portion from the belowground portion. The belowground parts were subsequently measured using a root scanner to determine the relevant root indices. After determination, the aboveground and belowground parts were placed in an oven at 105°C for 30 min until chlorosis, dried at 75°C to a constant weight, and subsequently weighed to determine the dry weights.

Plant photosynthetic indices, including the net photosynthesis rate (Pn), stomatal conductance (Gs), transpiration rate (Tr), and intercellular CO_2_ concentration (Ci), were quantified using an LI-6400 portable photosynthesis system. Physiological measurements of photosynthesis were taken on a sunny morning between 9 and 11am, 30 and 60 days after the initiation of drought stress. This was achieved using an open gas exchange system and red−blue light sources (LED) with a light intensity of 1500 cd. Three replicates were assessed per pot, and nine replicates were analysed for each treatment.

The plant physiological indices were determined by harvesting fresh sample leaves at 30 and 60 days after drought. The superoxide dismutase (SOD) activity was determined by the nitro blue tetrazolium method (Li et al., 2019). Catalase (CAT) activity was determined via the ultraviolet absorption method (Li et al., 2019). Malondialdehyde (MDA) content was determined by the thiobarbituric acid colorimetric method (Habib et al., 2020). The proline (Pro) content was determined by the ninhydrin colorimetric method (Habib et al., 2020). The soluble sugar content was determined by the anthrone method (Sharma et al., 2020). The soluble protein content was determined by the Kh Khao Maas Brilliant Blue G-250 colorimetric method (Ahmed et al., 2020).

### 2.3 Statistical analysis

The data are presented as the mean ± standard error of the mean (SEM) for samples with either three or nine replicates (Nine replications were carried out for plant height and plant photosynthetic indices, respectively, and three replications were carried out for the rest of the indicators). The data were statistically analysed using SPSS ver. 24.0 and Microsoft Excel 2021. Graphs were generated using GraphPad Prism 9. Clustering heatmaps were generated using R 4.3.1 The effects of different inoculation methods and cement contents on the physiological characteristics of the two plant species were investigated using one-way analysis of variance (ANOVA), two-way analysis of variance (ANOVA) and Pearson’s correlation analysis.

## 3 Results

### 3.1 Effects of AM fungi on plant growth characteristics

**Figure 1:**
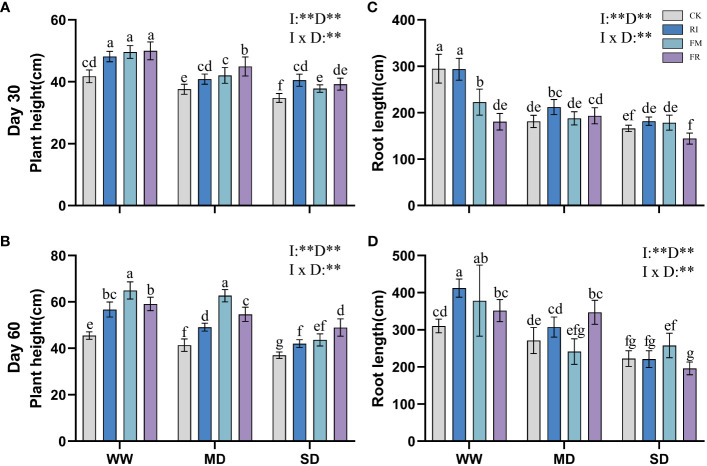
Effect of inoculation with AM fungi on plant height of *Festuca elata* under different drought levels.[**(A,C)** measured at 30 days, **(B, D)** measured at 60 days]. (WW, well-watered; MD, moderate drought; SD, severe drought. Based on one-way analysis of variance, different letters mean significant difference at 0.05 level; I, inoculation method; D, degree of drought; I × D, interaction between inoculation with AM fungi and drought stress. *p<0.05; **p<0.01; ns, not significant; CK, Control without inoculation; RI, Treatment with inoculation of *Rhizophagus intraradices* alone, FM, Treatment with inoculation of *Funneliformis mosseae* alone; FR,Treatment with mixed inoculation of RI and FM).

**Figure 2:**
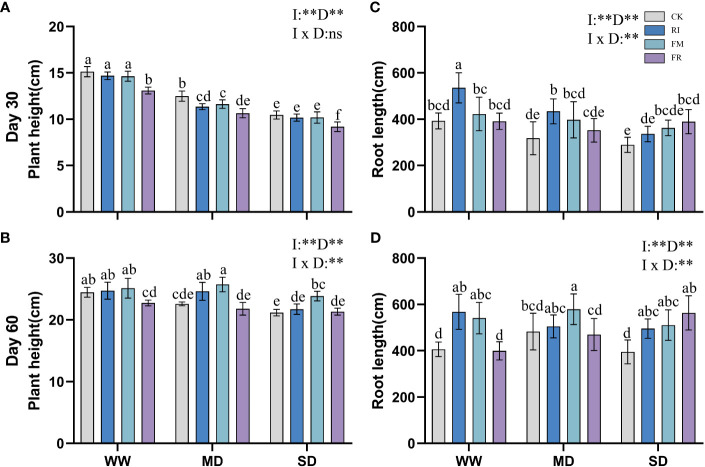
Effect of inoculation with AM fungi on plant height of *Cassia glauca* under different drought levels. [**(A, C)** measured at 30 days, **(B, D)** measured at 60 days]. (WW, well-watered; MD, moderate drought; SD, severe drought. Based on one-way analysis of variance, different letters mean significant difference at 0.05 level; I, inoculation method; D, degree of drought; I × D, interaction between inoculation with AM fungi and drought stress. *p<0.05; **p<0.01; ns, not significant; CK, Control without inoculation; RI, Treatment with inoculation of *Rhizophagus intraradices* alone, FM, Treatment with inoculation of *Funneliformis mosseae* alone; FR,Treatment with mixed inoculation of RI and FM).

In vegetation concrete, drought inhibits plant growth, leading to a decrease in plant height, root length, and biomass. This inhibitory effect becomes more pronounced with increasing severity of stress. Compared with that in the CK treatment, AM fungi inoculation significantly increased the plant height of *Festuca elata* (**Figure 1A**) (*p* < 0.05), and this increase became more evident over time, with the greatest improvement observed occurring at 60 days (51.59%) (**Figure 1B**). However, the increase in plant height in *Cassia glauca* (**Figure 2A**) plants caused by AM fungi inoculation was not significant, particularly at 30 days, when certain fungal groups exhibited suppression across all drought levels (FR). At 60 days (**Figure 2B**), the height of the FM plants slightly increased compared to that of the plants in the CK treatment, and this increase was significant at both drought levels (*p* < 0.05). The interaction effect between 30 days AM fungi inoculation and drought stress on *Cassia glauca* plant height wasn’t significant. However, 30 days and 60 days *Festuca elata* plant height, along with 60 days *Cassia glauca* showed significant impacts. Overall, AM fungi play a crucial role in alleviating drought-induced growth inhibition in *Festuca elata*. While AM fungi offer some benefits to *Cassia glauca* growth under drought, their impact appears less pronounced than that of *Festuca elata.*

Inoculation with AM fungi plays a certain role in promoting plant root growth, but the specific effects depend on the inoculation method and drought level. In the *Festuca elata* group, inoculation with AM fungi did not lead to greater growth in the WW treatment at 30 days (**Figure 1C**), but there was a certain increase in growth in the MD and SD treatment groups. Compared with those under CK conditions, the growth of plants inoculated with RI increased by 17.07% under MD conditions and 9.34% under SD conditions. At 60 days (**Figure 1D**), a different trend emerged. As drought increased, the ability of the AM fungi inoculation to promote root length gradually diminished. Under WW conditions, single inoculation with RI had the greatest increase (32.88%) compared to that in the CK treatment. In *Cassia glauca*, inoculation with AM fungi promoted the growth of plants under various drought levels. It performed better with single inoculation under WW and MD conditions, while FR inoculation resulted in greater growth under SD conditions. RI inoculation increased the root length by 36.35% and 39.79% under WW conditions, and FR inoculation increased the length under SD conditions, by of 34.74% and 42.68%, respectively (**Figures 2C, D**). A two-way ANOVA analysis showed that, the effect of inoculation with AM fungi on the growth of *Festuca elata* and *Cassia glauca* roots depends on the type of inoculation and drought severity.

**Figure 3:**
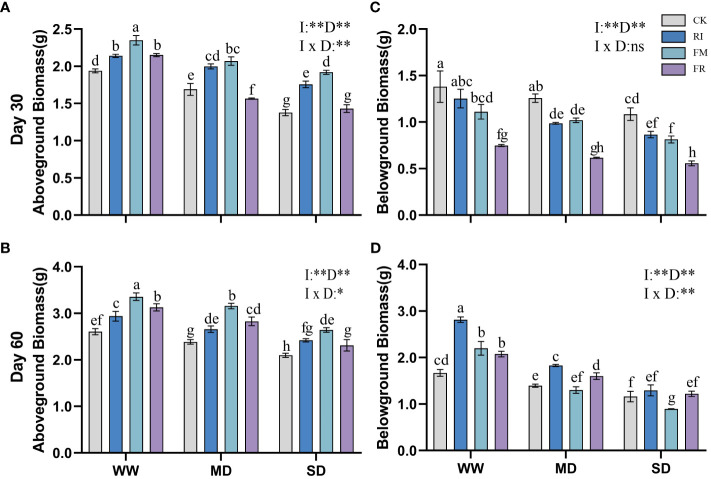
Effect of inoculation with AM fungi on biomass of *Festuca elata* under different drought levels. [**(A,C)** measured at 30 days, **(B,D)** measured at 60 days]. (WW, well-watered; MD, moderate drought; SD, severe drought. Based on one-way analysis of variance, different letters mean significant difference at 0.05 level; I, inoculation method; D, degree of drought; I × D, interaction between inoculation with AM fungi and drought stress. *p<0.05; **p<0.01; ns, not significant; CK, Control without inoculation; RI, Treatment with inoculation of *Rhizophagus intraradices* alone, FM, Treatment with inoculation of *Funneliformis mosseae* alone; FR,Treatment with mixed inoculation of RI and FM).

**Figure 4:**
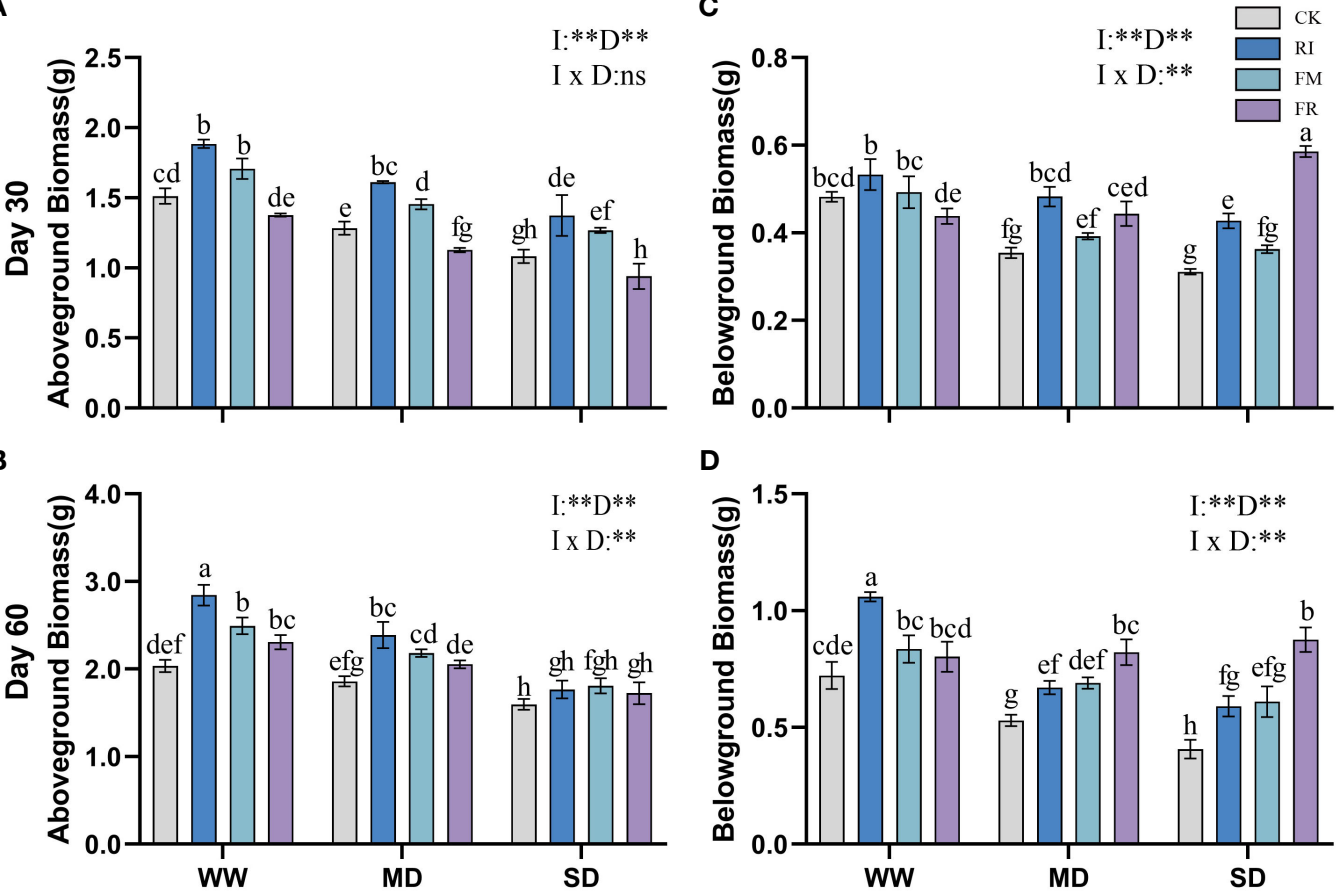
Effect of inoculation with AM fungi on biomass of *Cassia glauca* under different drought levels. [**(A, C)** measured at 30 days, **(B, D)** measured at 60 days]. (WW, well-watered; MD, moderate drought; SD, severe drought. Based on one-way analysis of variance, different letters mean significant difference at 0.05 level; I, inoculation method; D, degree of drought; I × D, interaction between inoculation with AM fungi and drought stress. *p<0.05; **p<0.01; ns, not significant; CK, Control without inoculation; RI, Treatment with inoculation of *Rhizophagus intraradices* alone, FM, Treatment with inoculation of *Funneliformis mosseae* alone; FR,Treatment with mixed inoculation of RI and FM).

AM fungi inoculation increased aboveground biomass. FM inoculation had the greatest effect on the accumulation of aboveground biomass in *Festuca elata*, with this increase becoming more pronounced as drought severity increased. At 30 days (**Figure 3A**), FM inoculation increased the aboveground biomass by 39.30% compared to that in the CK treatment. However, with worsening drought conditions, dual inoculation did not result in an increase trend in aboveground biomass. Nevertheless, over time, dual inoculation gradually led to a significant increase in aboveground biomass (**Figure 3B**). In the case of *Cassia glauca* (**Figures 4A, B**), RI inoculation resulted in greater aboveground biomass, and the increasing effect of dual inoculation was more pronounced in the later stages. It can be observed that in vegetation concrete, a single inoculation is more suitable for the accumulation of aboveground biomass in plants.

Inoculation with AM fungi inhibited the increase in the belowground biomass of *Festuca elata* (**Figure 3C**) after 30 days, with the most pronounced impact observed in the FR treatment, exhibiting reductions in belowground biomass of 45.83%, 50.97%, and 48.62% for the different drought levels compared with those in the CK treatment. However, at 60 days (**Figure 3D**), there was a certain promoting effect under WW and MD conditions, with the most notable increase observed in the RI. The increase in the belowground biomass of *Cassia glauca* (**Figures 4C, D**) caused by AM fungi inoculation was more significant at the later stage than at the earlier stage, and this increase gradually shifted from being stronger in the single-inoculation group to being stronger in the double-inoculation group. Non-significant interaction effects of inoculation method and drought level were observed in both *Festuca elata* and *Cassia glauca*, but both occurred only at 30 days. These findings show that belowground biomass responds differently to inoculation over time and under stress conditions, stressing the need for careful time and choosing the right inoculation approach for the best outcomes.

### 3.2 Effects of AM fungi on plant photosynthesis

**Figure 5:**
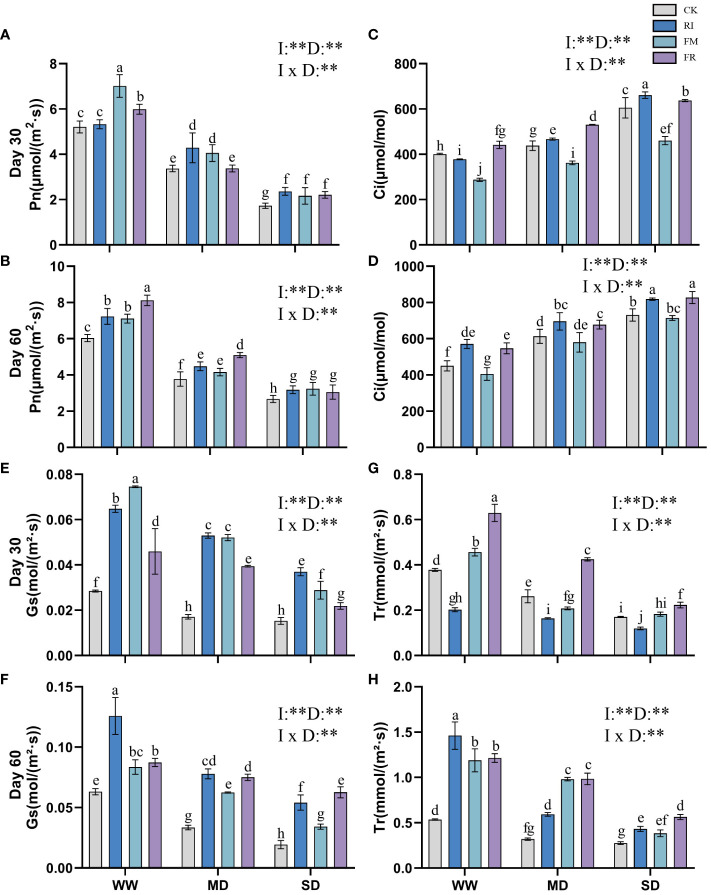
Effects of inoculation with AM fungi on the photosynthetic system of *Festuca elata* under different drought levels. [**(A, C, E, G)** measured at 30 days, **(B, D, F, H)** measured at 60 days]. (WW, well-watered; MD, moderate drought; SD, severe drought. Based on one-way analysis of variance, different letters mean significant difference at 0.05 level; I, inoculation method; D, degree of drought; I × D, interaction between inoculation with AM fungi and drought stress. *p<0.05; **p<0.01; ns, not significant; CK, Control without inoculation; RI, Treatment with inoculation of *Rhizophagus intraradices* alone, FM, Treatment with inoculation of *Funneliformis mosseae* alone; FR,Treatment with mixed inoculation of RI and FM).

**Figure 6:**
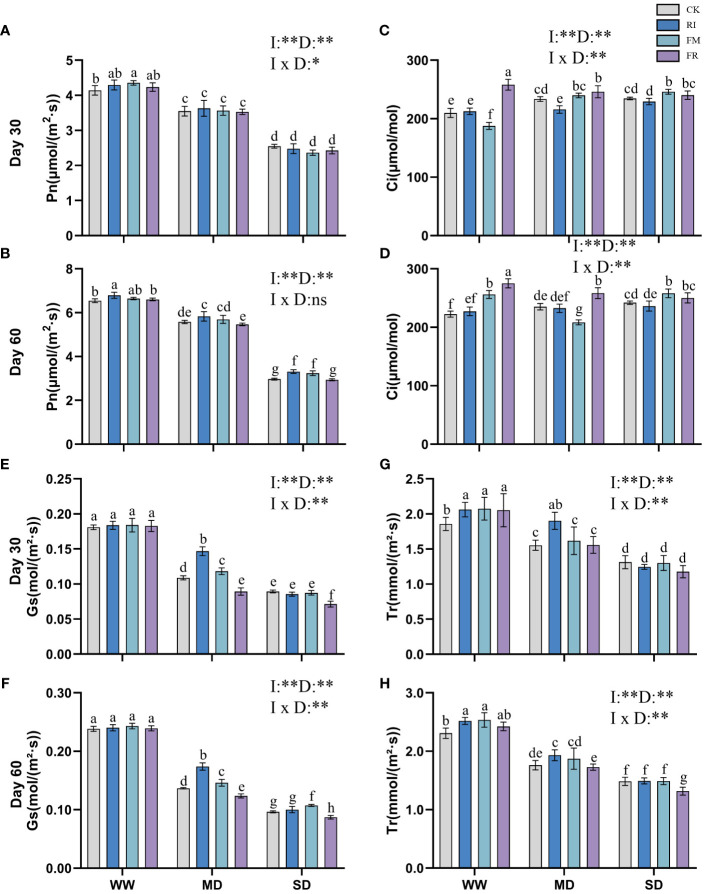
Effects of inoculation with AM fungi on the photosynthetic system of *Cassia glauca* under different drought levels. [**(A, C, E, G)** measured at 30 days, *(B, D, F, H)* measured at 60 days]. (WW, well-watered; MD, moderate drought; SD, severe drought. Based on one-way analysis of variance, different letters mean significant difference at 0.05 level; I, inoculation method; D, degree of drought; I × D, interaction between inoculation with AM fungi and drought stress. *p<0.05; **p<0.01; ns, not significant; CK, Control without inoculation; RI, Treatment with inoculation of *Rhizophagus intraradices* alone, FM, Treatment with inoculation of *Funneliformis mosseae* alone; FR,Treatment with mixed inoculation of RI and FM).

Increasing drought conditions lead to a reduction in the photosynthetic rate of plants. Inoculation with AM fungi somewhat increased the photosynthetic efficiency of *Festuca elata*. At 30 days (**Figure 5A**), the increase caused by dual inoculation was notably greater than that caused by single AM fungi inoculation (*p* < 0.05). After 60 days (**Figure 5B**), dual inoculation resulted in a greater Pn. However, the difference in Pn between the groups at different times after inoculation under SD conditions was not significant, but was significantly greater than that of the CK group (*p <*0.05). In *Cassia glauca*, the impact of AM fungi inoculation on the Pn was not significant after 30 days (**Figure 6A**). At 60 days (**Figure 6B**), the effect of RI inoculation was significantly greater than that of the CK treatment (*p* < 0.05), but the effect of FR inoculation was not significantly different from that of the CK treatment. Indeed, the findings underscore the species-specific responses to AM fungi inoculation and emphasise the importance of considering both the plant species and the type and duration of inoculation when assessing the potential benefits of AM fungi in enhancing photosynthetic efficiency under drought conditions.

The Ci of *Festuca elata* increased with increasing drought stress severity (**Figures 5C, D**). In *Cassia glauca*, a similar trend to that observed in *Festuca elata* was noted at 30 days (**Figure 6C**). Single inoculation with FM resulted in a reduction in Ci in *Festuca elata* (**Figures 5C, D**), while the other two types of inoculation elevated Ci to a certain extent. Dual inoculation of *Cassia glauca* yielded the highest Ci (**Figures 6C, D**). These findings underscore the differential responses of Ci to AM fungi inoculation across plant species and highlight the potential for dual inoculation to modulate Ci under drought stress conditions.

The Gs of *Festuca elata* decreased with intensified drought stress, while inoculation with AM fungi significantly increased the Gs (*p* < 0.05). At 30 days (**Figure 5E**), single inoculation resulted in greater a increase, and at 60 days (**Figure 5F**), this advantage was maintained for single inoculation except under SD conditions. In *Cassia glauca*, inoculation with AM fungi significantly differed under MD conditions. Compared with the CK, single inoculation increased Gs, while dual inoculation had the opposite effect (**Figures 6E, F**). These results highlight the nuanced response of Gs to AM fungi inoculation across different plant species and drought stress levels.

With increasing drought stress, the Tr of plant leaves in vegetation concrete tended to decrease trend. At 30 days in *Festuca elata*, the plants in the CK and FR treatments maintained a greater transpiration rate, especially under drought conditions (**Figure 5G**). After 60 days (**Figure 5H**), the CK treatment did not maintain its dominance, and the AM fungus-inoculated groups exhibited greater Tr values than did the CK group. The dual inoculation treatment resulted in greater values being maintained under drought conditions. Only the RI treatment in *Cassia glauca* maintained a greater transpiration rate than did the CK at all three levels (**Figures 6G, H**). In conclusion, while increasing drought stress generally led to a decrease in transpiration rates, the inoculation of AM fungi, particularly through dual inoculation, emerged as a significant factor in sustaining or even enhancing transpiration rates.

Incorporating two-way analysis of variance, it was observed that both inoculation with AM fungi and drought stress, as well as their interaction, significantly influenced various photosynthetic parameters in *Festuca elata*. However, in *Cassia glauca*, the interaction effect was only significant at 30 days but not at 60 days. This discrepancy may be attributed to the stronger correlation between the overall photosynthetic performance of *Cassia glauca* and morphological leaf traits such as leaf area and leaf number.

### 3.3 Effects of AM fungi on plant biochemical indicators

#### 3.3.1 Antioxidant enzyme activity

**Figure 7:**
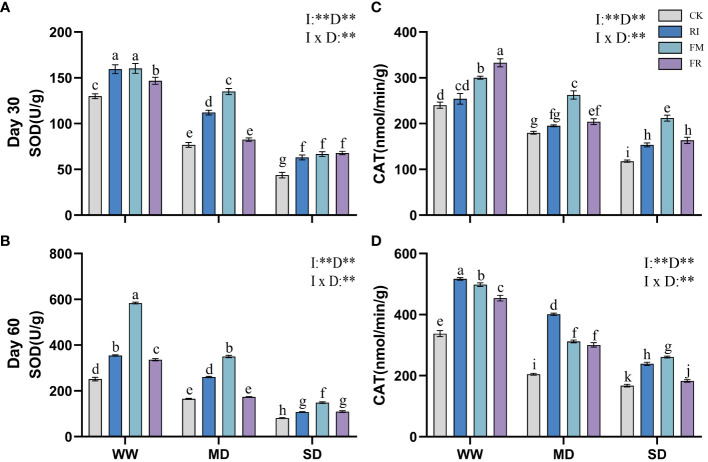
Effects of inoculation with AM fungi on the antioxidant enzyme activity of *Festuca elata* under different drought levels. [**(A, C)** measured at 30 days, **(B, D)** measured at 60 days]. (WW, well-watered; MD, moderate drought; SD, severe drought. Based on one-way analysis of variance, different letters mean significant difference at 0.05 level; I, inoculation method; D, degree of drought; I × D, interaction between inoculation with AM fungi and drought stress. *p<0.05; **p<0.01; ns, not significant; CK, Control without inoculation; RI, Treatment with inoculation of *Rhizophagus intraradices* alone, FM, Treatment with inoculation of *Funneliformis mosseae* alone; FR,Treatment with mixed inoculation of RI and FM).

**Figure 8:**
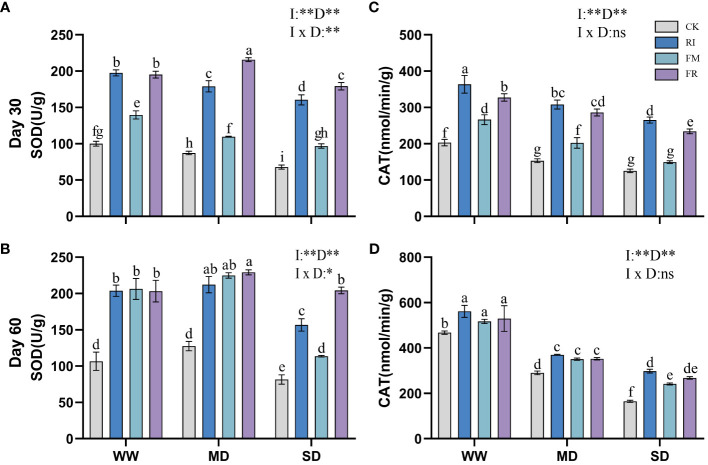
Effects of inoculation with AM fungi on the antioxidant enzyme activity of *Cassia glauca* under different drought levels. [**(A, C)** measured at 30 days, **(B, D)** measured at 60 days]. (WW, well-watered; MD, moderate drought; SD, severe drought. Based on one-way analysis of variance, different letters mean significant difference at 0.05 level; I, inoculation method; D, degree of drought; I × D, interaction between inoculation with AM fungi and drought stress. *p<0.05; **p<0.01; ns, not significant; CK, Control without inoculation; RI, Treatment with inoculation of *Rhizophagus intraradices* alone, FM, Treatment with inoculation of *Funneliformis mosseae* alone; FR,Treatment with mixed inoculation of RI and FM).

Drought caused a decrease in leaf SOD activity. Inoculation with AM fungi significantly increased leaf SOD activity. In *Festuca elata*, single inoculation increased leaf SOD activity compared to that in the CK treatment (**Figures 7A, B**). Among the different drought treatment conditions, FM resulted in the highest enzyme activity. The lowest level of SOD activity in *Cassia glauca* leaves occurred under SD conditions, and the highest level occurred under MD conditions (**Figures 8A, B**). However, the lowest leaf SOD activity was found under the FM treatment, and the maximum value occurred under the FR treatment, which was different from that observed for *Festuca elata*. The activity of SOD enzyme in both *Festuca elata* and *Cassia glauca* was significantly influenced by the inoculation of AM fungi, drought level, and their interaction. In essence, while drought stress generally reduced leaf SOD activity, inoculation with AM fungi notably increased SOD activity. Interestingly, the response varied between species, with *Festuca elata* showing elevated SOD activity with single inoculation, particularly under FM treatment, whereas *Cassia glauca* exhibited contrasting patterns, indicating the intricate interplay between species-specific responses and environmental conditions in shaping antioxidant defence mechanisms.

Drought caused a decrease in leaf CAT activity, whereas AM fungi inoculation effectively increased CAT activity in plant leaves (**Figures 7C, D**). In *Festuca elata* compared to the CK treatment, the RI treatment showed a maximum increase in CAT of 95.78% at 60 days under MD conditions (**Figure 7D**). Overall, single inoculation resulted in increased CAT activity. However, in *Cassia glauca*, although the RI treatment had greater effects on for CAT activity than the FR treatment, the FM treatment did not yield better results than the FR treatment (**Figures 8C, D**). This finding is in contrast to the pattern observed in the *Festuca elata*. In *Festuca elata*, CAT activity is significantly influenced by the inoculation of AM fungi, drought level, and their interaction. Conversely, in *Cassia glauca*, CAT activity is only significantly affected by the inoculation of AM fungi and drought level, with no significant interaction effect In summary, while AM fungi inoculation consistently enhances CAT activity in both *Festuca elata* and *Cassia glauca*, the differential responses between species underscore the complexity of plant-fungi interactions and highlight the importance of considering species-specific characteristics in optimising strategies for enhancing antioxidant defence mechanisms under drought conditions.

#### 3.3.2 Osmotic adjustment substances

**Figure 9:**
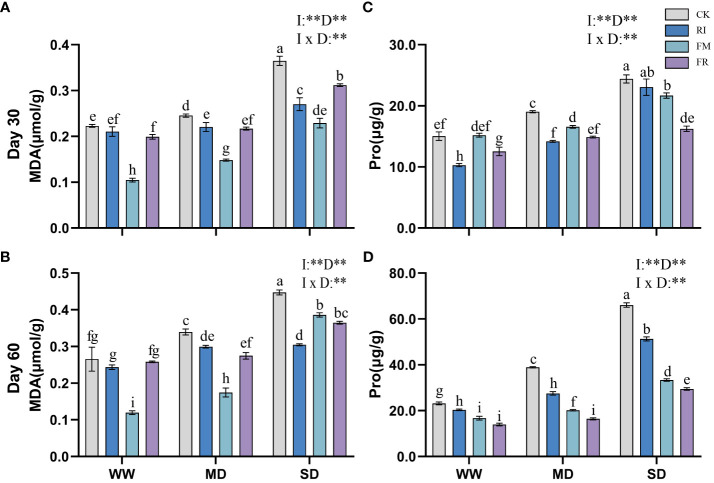
Effects of inoculation with AM fungi on MDA and Pro content of *Festuca elata* under different drought levels.[**(A, C)** measured at 30 days, **(B, D)** measured at 60 days]. (WW, well-watered; MD, moderate drought; SD, severe drought. Based on one-way analysis of variance, different letters mean significant difference at 0.05 level; I, inoculation method; D, degree of drought; I × D, interaction between inoculation with AM fungi and drought stress. *p<0.05; **p<0.01; ns, not significant; CK, Control without inoculation; RI, Treatment with inoculation of *Rhizophagus intraradices alone*, FM, Treatment with inoculation of *Funneliformis mosseae* alone; FR,Treatment with mixed inoculation of RI and FM).

**Figure 10:**
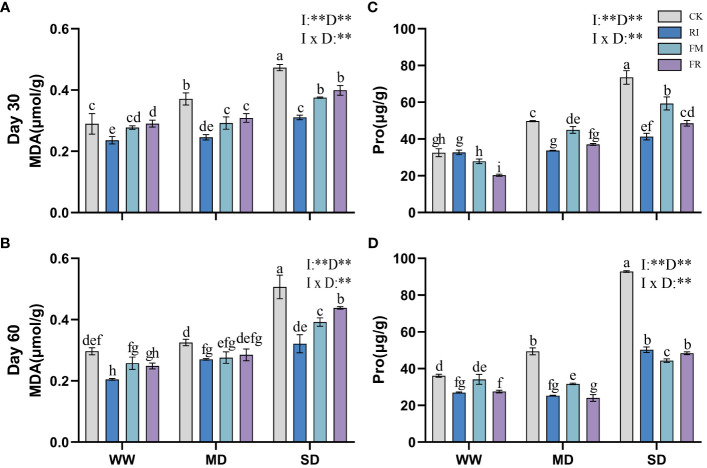
Effects of inoculation with AM fungi on MDA and Pro content of *Cassia glauca* under different drought levels. [**(A, C)** measured at 30 days, **(B, D)** measured at 60 days]. (WW, well-watered; MD, moderate drought; SD, severe drought. Based on one-way analysis of variance, different letters mean significant difference at 0.05 level; I, inoculation method; D, degree of drought; I × D, interaction between inoculation with AM fungi and drought stress. *p<0.05; **p<0.01; ns, not significant; CK, Control without inoculation; RI, Treatment with inoculation of *Rhizophagus intraradices* alone, FM, Treatment with inoculation of *Funneliformis mosseae* alone; FR,Treatment with mixed inoculation of RI and FM).

Drought stress caused an increase in the MDA content in the plant leaves. Inoculation with AM fungi significantly reduced leaf MDA accumulation (*p* < 0.05). Under most conditions, FM treatment resulted in the lowest MDA content in *Festuca elata* (**Figures 9A, B**). At 60 days (**Figure 9B**), under WW conditions, the FM treatment resulted in a 55.01% reduction in the accumulation of MDA compared with the CK treatment, but under SD conditions, the RI treatment resulted in an even lower MDA content. Overall, single inoculation was more effective at reducing the accumulation of leaf MDA in *Festuca elata*. In *Cassia glauca*, compared with the CK treatment, the RI treatment had a greater effect on reducing the MDA content by 36.64% under 60-day SD conditions (**Figures 10A, B**). Dual inoculation was less effective at reducing MDA accumulation, which was consistent with the findings in the *Festuca elata* group. In both *Festuca elata* and *Cassia glauca*, dual inoculation is more beneficial in reducing the accumulation of MDA content.”

The Pro content in plants increased significantly under drought conditions. However, Pro accumulation in leaves could be significantly reduced by inoculation with AM fungi. In *Festuca elata*, the inhibitory effects of inoculation with RI or FR on Pro accumulation were most significant within the first 30 days of drought stress (**Figure 9C**). At 60 days (**Figure 9D**), the inhibitory effect of FR inoculation on Pro production was more significant. In contrast, *Cassia glauca* also exhibited greater inhibition of Pro accumulation within 30 days in response to the RI and FR treatments (**Figure 10C**). In contrast to *Festuca elata*, FM inoculation had the greatest inhibitory effect on Pro accumulation under severe drought conditions after 60 days (**Figure 10D**). *Festuca elata* and *Cassia glauca*, both exhibit significant effects on MDA and Pro content due to the inoculation of AM fungi, drought level, and their interaction.

**Figure 11:**
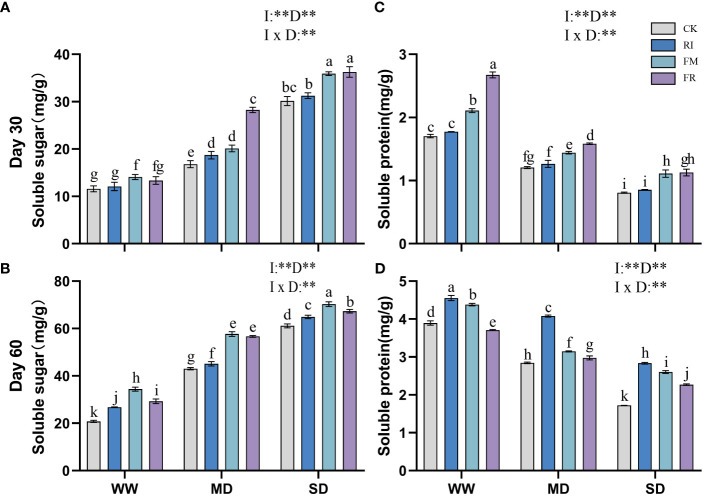
Effects of inoculation with AM fungi on soluble sugar and soluble protein content of *Festuca elata* under different drought levels. [**(A, C)** measured at 30 days, **(B, D)** measured at 60 days]. (WW, well-watered; MD, moderate drought; SD, severe drought. Based on one-way analysis of variance, different letters mean significant difference at 0.05 level; I, inoculation method; D, degree of drought; I × D, interaction between inoculation with AM fungi and drought stress. *p<0.05; **p<0.01; ns, not significant; CK, Control without inoculation; RI, Treatment with inoculation of *Rhizophagus intraradices* alone, FM, Treatment with inoculation of *Funneliformis mosseae* alone; FR,Treatment with mixed inoculation of RI and FM).

**Figure 12:**
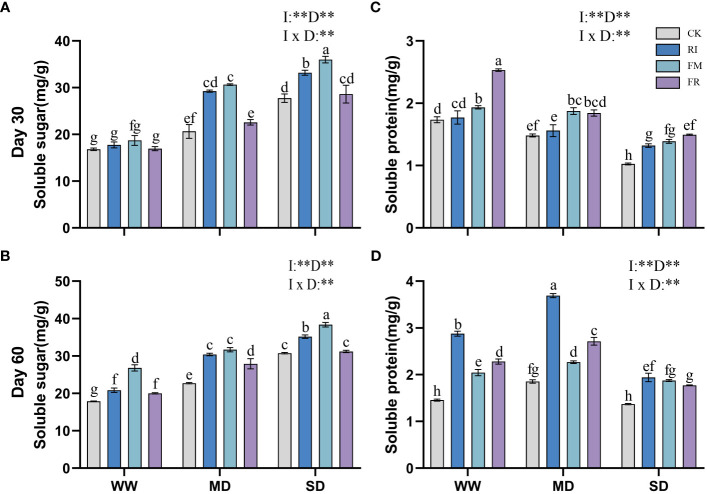
Effects of inoculation with AM fungi on soluble sugar and soluble protein content of *Cassia glauca* under different drought levels. [**(A, C)** measured at 30 days, **(B, D)** measured at 60 days]. (WW, well-watered; MD, moderate drought; SD, severe drought. Based on one-way analysis of variance, different letters mean significant difference at 0.05 level; I, inoculation method; D, degree of drought; I × D, interaction between inoculation with AM fungi and drought stress. *p<0.05; **p<0.01; ns, not significant; CK, Control without inoculation; RI, Treatment with inoculation of *Rhizophagus intraradices* alone, FM, Treatment with inoculation of *Funneliformis mosseae* alone; FR,Treatment with mixed inoculation of RI and FM).

Drought stress caused an increase in the soluble sugar content in the leaves, and AM fungi inoculation also increased in the soluble sugar content. In *Festuca elata*, the leaves of the FR group had a greater soluble sugar content at 30 days (**Figure 11A**), which was 68.22% greater than that of the CK group under MD conditions. However, the soluble sugar content of plants in the FM group was greater than that of plants in the FR group with increasing duration of stress (**Figure 11B**). In *Cassia glauca*, the single inoculation group consistently presented a greater increase in soluble sugar content than did the dual inoculation group throughout all the experimental periods (**Figures 12A, B**). In addition, within the single inoculation treatment, FM treatment had a more pronounced effect. The soluble sugar content in both *Festuca elata* and *Cassia glauca* are significantly influenced by the inoculation of AM fungi, drought level, and their interaction. These findings highlight the dynamic response of soluble sugar accumulation to both drought stress and AM fungi inoculation, suggesting potential strategies for enhancing stress tolerance in plants.

Drought led to a decrease in soluble protein content in plant leaves, but at 60 days (**Figure 12D**), *Cassia glauca* plants under MD conditions presented a maximum soluble protein content. Inoculation with AM fungi increased the soluble protein content in the plant leaves. In *Festuca elata*, dual inoculation had a greater effect at 30 days (**Figure 11C**), while single inoculation had a greater effect at 60 days (**Figure 11D**). A similar trend was observed for *Cassia glauca*. Dual inoculation resulted in a greater soluble protein content at 30 days, and at 60 days, single inoculation with RI had stronger effects (**Figures 12C, D**). The soluble protein content, along with soluble sugar, is consistently significantly influenced by the inoculation of AM fungi, drought level, and their interaction. These findings underscore the dynamic interplay between soluble protein accumulation, drought stress, and AM fungi inoculation, suggesting avenues for enhancing protein synthesis and bolstering plant stress tolerance.

### 3.4 PCA and clustering analysis

**Figure 13:**
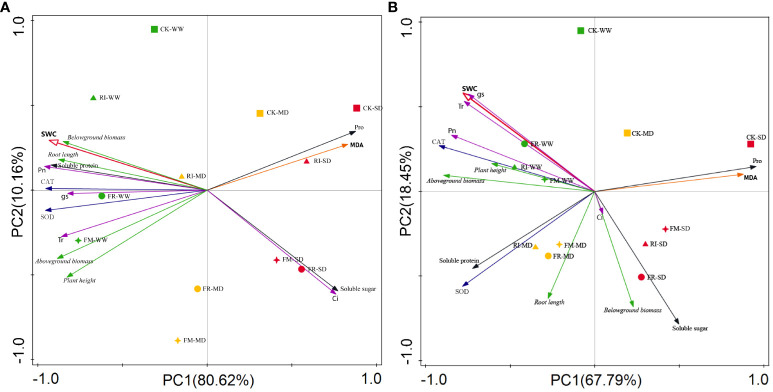
PCA of the effects of inoculation with AM fungi on various indicators of two plants under different drought levels. (**A**, **B** represent *Festuca elata* and *Cassia glauca* respectively.).

We conducted a principal component analysis (PCA) to identify the main factors influencing the growth of *Festuca elata* and *Cassia glauca*. As shown in **Figure 13A**, in *Festuca elata*, Pro, MDA, soluble sugar, Ci, and soil water content (SWC) exhibited strong negative correlations, while the other indicators exhibited positive correlations with SWC. Among them, biomass, soluble protein, and Pn were strongly positively correlated with SWC. Taken together, these findings suggest that, under drought stress, *Festuca elata* plants experience a significant decrease in biomass, soluble protein, and Pn, while the leaf Pro, MDA, soluble sugar content, and Ci increase. As shown in **Figure 13B**, in *Cassia glauca*, Pro, MDA, soluble sugar, and belowground biomass strongly negatively correlated with SWC, while Pn, Gs, Tr, CAT, and SWC strongly positively correlated with SWC. Taken together, these findings indicate that during drought stress in *Cassia glauca*, the photosynthetic system is affected first, and at the same time, the contents of Pro, MDA, soluble sugars, and other components increase. Under the same level of drought stress, in both plant species, the CK treatment was positioned closest to the positive direction of PC1, which was opposite to the direction of SWC. This indicates that the plants in the CK group experienced more severe drought stress, and inoculation with AM fungi helps improve the drought resistance of the plants in the vegetation concrete.

**Figure 14:**
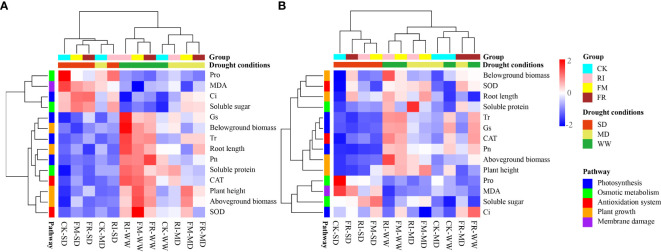
Cluster analysis of inoculation with AM fungi on various indicators of two plants under different drought levels. (**A**, **B** represent *Festuca elata* and *Cassia glauca* respectively.).

According to the heatmap clustering analysis (**Figure 14**), we found clear clustering patterns. Under drought treatment conditions, the plants in the SD group exhibited consistent physiological responses, while those in the WW and MD treatment groups exhibited similar physiological responses. This finding suggested that drought within the vegetation zone influenced the physiological responses of the plants to some extent. We observed a significant increase in the levels of Pro and MDA under drought stress conditions, indicating a pronounced physiological response in plants under drought pressure and a significant reduction in plant height, biomass and photosynthetic efficiency, indicating the adverse effects of drought on plant growth and metabolism.

Conversely, the AM fungus-inoculated group presented greater photosynthetic efficiency, biomass and antioxidant enzyme activity. This finding suggested a potential beneficial role for AM fungi in increasing plant drought resistance. Notably, the plants in the control group exhibited greater accumulation of proline and malondialdehyde, suggesting that plants may be exposed to more severe oxidative stress without AM fungal inoculation. This further supports the idea that AM fungi inoculation increases plant drought resistance and mitigates stress responses under drought conditions.

## 4 Discussion

Drought is recognised as one of the most severe abiotic stresses worldwide, and a plant’s ability to tolerate drought is crucial for its survival and growth in arid environments. Although AM fungi have been shown to improve drought tolerance in many plant species, the effectiveness of different fungal strains in targeting different plants in complex ecosystems is still unknown (Li et al., 2019; Sheteiwy et al., 2021a; Nacoon et al., 2022). Most related studies have focused primarily on applying drought stress to natural soils. However, there is still limited knowledge on the effects of inoculating plants with AM fungi in vegetation concrete, which is used as a substrate for berms and has distinct physicochemical properties that differ from those of natural soils. In this study, we simulated three drought levels in vegetation concrete and selected two pioneer plants for slope protection to investigate whether and how AM fungi inoculation could positively influence plant drought resistance within a sloped environment. Furthermore, we aimed to assess whether drought stress affects plant adaptation to drought and whether it can increase the quality of drought resistance for slope greening.

Many studies have shown that plants that form a good symbiotic structure with AM fungi tend to have greater biomass, plant height and root length (Cheng et al., 2021; Nacoon et al., 2022; Yang Y M et al., 2023). In our study, the inoculation of vegetation concrete with AM fungi improved plant growth performance and increased plant biomass. Compared with the plants that did not form symbiotic structures, the plants that formed symbiotic structures performed better to varying degrees, with increases in plant height, root length, aboveground biomass and belowground biomass; however, this phenomenon was more pronounced at 60 days, and the greatest increases were 51.59%, 42.68%, 39.71% and 115.46%, respectively. The reason for this phenomenon may be that AM fungi form symbiotic structures with plant roots; increase the expression of genes related to the root epidermis, xylem, phloem and root hairs; stimulate the growth of primary and lateral roots of mycorrhizal plants; increase the size of mycorrhizal plants; and increase the effective absorptive area of the root system, which in turn improves the water uptake capacity (Liu H et al., 2023; Xi et al., 2022). This symbiotic relationship activates the physiological regulatory mechanisms of plants and increases their resistance to environmental stresses. AM fungi can absorb nutrients and minerals that are difficult for plants to utilise directly, especially phosphate and nitrogen, through their mycelia and deliver them to plants, which can promote the synthesis of amino acids needed for protein synthesis, thus promoting the growth and development of plants (Iffis et al., 2017; Shu et al., 2022; Xi et al., 2022). The presence of AM fungi can also regulate the quality of the soil around plants, and on the other hand, AM fungi can regulate the nutrient ratios of the soil surrounding plants, improving plant nutrient status (Chareesri et al., 2020; Jia et al., 2021; Liu H et al., 2023). Moreover, the data showed that the different AM fungi had different effects on plant growth strategies. In *Festuca elata*, AM fungi promoted plant height slightly more obviously than did the other fungal species, and the effect of single inoculation was greater. In *Cassia glauca*, AM fungi tended to increase root length. However, we found an interesting phenomenon in the experiments. In *Cassia glauca*, although AM fungal inoculation did not significantly increase plant height, it tended to increase the number of leaves, which may be useful for obtaining more efficient photosynthesis. Moreover, the biomass of the inoculated plants increased regardless of the drought level, which further confirmed the important role of AM fungi in promoting plant growth and material accumulation.

Water is one of the most important factors limiting plant photosynthesis, Drought leads to the formation of a hydraulic gradient between the leaf and the soil, which accelerates the development of leaf water deficit by reducing leaf expansion pressure further reducing stomatal conductance, and weakening gas exchange with the atmosphere. The efficiency of photosynthesis is inhibited by this behaviour, which ultimately hinders plant growth (Huang et al., 2021; Yang Y M et al., 2023; Gui et al., 2021). In this study, the photosynthetic rate of plant leaves decreased with increasing drought, and plants inoculated with AM fungi had greater Pn and Gs values (the greatest increase was 36.7% and 210.08% respectively compared to those in the CK treatment), which provided more efficient photosynthetic and gas exchange pathways for the plants. These findings also indicated that the degree of water deficit was less harmed in plants inoculated with AM fungi (Cheng et al., 2021; Li et al., 2019; Huang et al., 2021). Therefore, plants inoculated with AM fungi have greater Ci, which corresponds to changes in Gs, more open stomata, and less restriction of CO_2_ entry (Huang et al., 2020; Duan et al., 2021; Yang Y M et al., 2023). Although many studies have shown that AM fungi modulate the stomatal switching of plants in response to drought, the degree of stomatal opening also verifies the degree to which plants are subjected to drought stress, and the greater stomatal conductance after inoculation with AM fungi suggests that plants are less persecuted by drought and do not need to be upregulated to close their stomata (Abdalla et al., 2023). In terms of the transpiration rate, plants inoculated with AM fungi showed a partial decrease in transpiration rate after 30 days of drought (0.89%~37.37%), which could be attributed to the formation of the symbiotic structure of AM fungi entering the acclimatisation stage, wherein the plants still need to provide part of the photosynthesis-synthesised carbohydrates to the AM fungi in exchange for essential nutrients and minerals for growth and then need to reduce transpiration to minimise the loss of water and maintain stable growth (Bárzana et al., 2012; Sun et al., 2021). However, the increase in the transpiration rate after 60 days (0.37%~208.61%) suggested that the plants may have developed more efficient water uptake, utilisation and transport mechanisms, at which point the transpiration rate was no longer the limiting factor and did not need to be reduced to meet growth requirements (Duddridge et al., 1980; Bárzana et al., 2012). This suggests that plants inoculated with AM fungi may be more drought tolerant, and that AM fungi can regulate the water use efficiency of plants in the early stages, reducing water loss and damage caused by abiotic stresses.

As drought intensifies, plants accumulate large amounts of reactive oxygen species (ROS), such as superoxide anion radical (O^2-^), hydrogen peroxide (H_2_O_2_) and hydroxyl radical (OH) (Huang et al., 2020; Liu H et al., 2023; Sun et al., 2021). The excessive accumulation of these ROS induced by drought stress leads to oxidative damage in plants, causing lipid peroxidation of cell membranes, leading to protein denaturation, nucleic acid damage, oxidation of carbon compounds, pigment catabolism, and induction of programmed cell death. To avoid damage caused by high concentrations of ROS, plants reduce damage under drought conditions through the antioxidant enzyme system (Huang et al., 2020; Li et al., 2022; Tsuji et al., 2022). In the present study, the SOD and CAT activities in the AM-inoculated group were significantly greater than those in the CK treatment (the greatest increases were 164.14% and 111.59%, respectively compared to those in the CK treatment), suggesting that the former has greater drought resistance and suffers less oxidative damage. The MDA content in plant tissues is often used as an important indicator of ROS homeostasis and plasma membrane damage in plant tissues (Li et al., 2022). In the present study, the MDA content in plant leaves increased significantly with worsening drought, but the accumulated MDA content in the plant leaves of the AM inoculation group was lower than that in the CK treatment (2.79~55.01%). These findings indicated that inoculation with AM fungi could reduce the oxidative damage caused by drought by increasing the activity of antioxidant enzymes, which subsequently reduced the accumulation of MDA. The data showed that the trend for the Pro content was similar to that for MDA content, and drought increased the Pro content in leaves, while inoculation with AM fungi reduced the accumulation of Pro (5.53~57.74%). It is commonly believed that plants accumulate Pro to increase drought resistance to reduce drought damage under water deficit conditions (Thangaraj et al., 2022; Wang et al., 2023). The low Pro accumulation in the inoculated group suggested that these plants were less exposed to drought and did not require excessive proline accumulation to increase drought resistance. During drought, plants also alter osmotic potential by increasing the synthesis of osmoregulatory substances to maintain normal growth and metabolism, but drought stress inhibits photosynthesis in plants, leading to a decrease in carbohydrate yield (Li et al., 2019, 2022; Yang Y M et al., 2023). In our study, AM fungal inoculation also significantly increased soluble sugars and soluble proteins in plant leaves (the greatest increases were 68.22% and 97.54%, respectively compared to those in the CK treatment). The reason for this phenomenon is that plants accumulate soluble sugars in their leaves in response to environmental stresses, a behaviour in which both maintain osmotic balance to reduce drought persecution and serve as a potential source of energy (Nacoon et al., 2022; Tsuji et al., 2022; Wang et al., 2023). During drought, plants regulate protein synthesis, inducing an increase in specific proteins with functions such as antioxidant, stress response, and signalling regulation, thereby increasing drought tolerance (Bárzana et al., 2012; Wang et al., 2023). Higher levels of soluble sugars and soluble proteins are also evidence that inoculation with AM fungi provides plants with greater drought tolerance.

## 5 Conclusions

We first investigated the effect of the incorporation of exogenous AM fungi into vegetation concrete on the drought tolerance of slope plants. Under drought conditions, inoculation with or without AM fungi impaired plant growth and photosynthesis. Inoculation of vegetation concrete with AM fungi increased plant height, root length and biomass. The Pn, Gs and Tr of *Festuca elata* were somewhat elevated after inoculation with AM fungi (the greatest increases were 36.72%, 210.08%, and 66.41%, respectively compared to those in the CK treatment), whereas the Gs and Tr of *Cassia glauca* increased only under MD conditions. Inoculation with AM fungi can promote drought tolerance by increasing the activity of antioxidant enzymes and the accumulation of osmoregulatory substances in plants (4.76~164.14%, 0.79~99.14%). Single inoculation is beneficial to the growth of aboveground biomass, and dual inoculation is more beneficial to the growth of belowground biomass. In the early stage of slope ecological restoration, higher aboveground biomass is more conducive to the regreening effect of slope ecological restoration projects, but the presence of plant roots in slope ecological restoration projects is one of the most important factors for reducing soil erosion and improving the stability of slopes. Therefore, in slope ecological restoration projects, the texture of slopes and sowing plants should be considered by different means. Gentle slopes or herbaceous plants are dominated by slope ecological restoration projects Slow slopes or herbaceous plant-based slope ecological restoration projects should be based on single inoculation methods, and steep slopes or shrub-based slope ecological restoration projects should be based on dual inoculation methods. The stability of slopes is a key factor in improving the sustainability of slopes; therefore, in subsequent studies, attention should be given to the effects of inoculation with AM fungi on the modulation of plant root morphology and the structure and erosion resistance of root-soil complexes to assess their contribution to the stability of ecological restoration projects on slopes.

## Data availability statement

The raw data supporting the conclusions of this article will be made available by the authors, without undue reservation.

## Author contributions

SG: Conceptualization, Data curation, Formal analysis, Writing – original draft. LX: Formal analysis, Resources, Writing – review & editing. DX: Funding acquisition, Writing – review & editing. ML: Resources, Writing – review & editing. WX: Funding acquisition, Project administration, Writing – review & editing. LL: Funding acquisition, Supervision, Writing – review & editing.
